# HPV Self-Sampling for Cervical Cancer Screening in Under-Screened Saskatchewan Populations: A Pilot Study

**DOI:** 10.3390/curroncol31080317

**Published:** 2024-07-26

**Authors:** Erin Vink, Gabriella Antaya, Camille Hamula, Carla Holinaty, Jessica Minion, Gregory R. Pond, Sabryna McCrea, Lynn Dwernychuk, Holly Graham, Gordon Broderick, Mary Kinloch, Jennifer Brown Broderick

**Affiliations:** 1 College of Medicine, University of Saskatchewan, Saskatoon, SK S7N 5A2, Canada; ywp941@usask.ca (E.V.); gabby.antaya@usask.ca (G.A.); srm717@usask.ca (S.M.); 2 Department of Laboratory Medicine and Pathology, College of Medicine University of Saskatchewan, Saskatoon, SK S7N 0W8, Canada; camille.hamula@saskhealthauthority.ca (C.H.); jessica.minion@saskhealthauthority.ca (J.M.); mary.kinloch@saskhealthauthority.ca (M.K.); 3 Department of Academic Family Medicine, University of Saskatchewan, Saskatoon, SK S7N 3Y5, Canada; carla.holinaty@usask.ca; 4 Department of Oncology, Faculty of Health Sciences, McMaster University, Hamilton, ON L8V 5C2, Canada; gpond@mcmaster.ca; 5 Saskatchewan Cancer Agency, Saskatoon, SK S7N 4H4, Canada; lynn.dwernychuk@saskcancer.ca; 6 Department of Psychiatry, College of Medicine, University of Saskatchewan, Saskatoon, SK S7K 2Z4, Canada; holly.graham@usask.ca; 7 Vaccine and Infectious Disease Organization (VIDO), Saskatoon, SK S7N 5E3, Canada; gordon.broderick@usask.ca; 8 Department of Oncology, College of Medicine, University of Saskatchewan, Saskatoon, SK S7N 4H4, Canada

**Keywords:** HPV, cervical cancer screening, self-sampling

## Abstract

Of all cancers in female Canadians, the most rapidly increasing incidence is that of cervical cancer. The objective of this pilot study was to assess how HPV self-sampling might improve cervical cancer screening participation in both urban and rural settings in Saskatchewan, one of the most sparsely populated provinces in Canada. Study groups consisted of *n* = 250 participants to whom self-swabbing kits were mailed with instructions and *n* = 250 participants to whom kits were handed out in 6 urban and rural clinics. The inclusion criteria selected subjects aged 30–69 years who were Saskatchewan residents for at least 5 years with valid health coverage, had a cervix, and had no record of cervical cancer screening in 4 years. The returned samples were analyzed for specific HPV strains using the Roche Molecular Diagnostics Cobas 4800^®^ System. The overall response rate was ~16%, with the response to the handout distribution being roughly double that of the mailout. While HPV positivity did not differ across the distribution groups, participants at a specific inner-city clinic reported significantly higher positivity to at least one HPV strain as compared to any other clinic and all mailouts combined. For this high-risk population, in-person handout of self-sampling kits may be the most effective means of improving screening.

## 1. Introduction

Cervical cancer in Canada has been increasing 3.7% per year since 2015, a rate faster than all other cancers in females in the same timeframe. In 2023, there were 1550 cases in Canada, with 440 individuals dying of the disease [1]. The province of Saskatchewan reported 45 women with new diagnoses of cervical cancer in 2020 alone, with almost half of these women dying of this illness [2]. In 2024, the Canadian Cancer Society estimates 60 cervical cancer diagnoses with 15 deaths, despite cervical cancer being largely preventable through improved access to screening and HPV (Human papillomavirus) vaccination [1].

At the time of this study, cervical cancer screening guidelines in Saskatchewan recommend that women aged 21–69 be encouraged to visit to a physician or other health care professional for PAP (Papanicolaou) testing. While these guidelines are similar in other provinces, the percentage of eligible women undergoing recommended screening was lowest in Saskatchewan at 49% [3] and well below 80%, the target set by the Pan-Canadian Cervical Cancer Screening Network [4]. Screening adherence is even lower in underserved populations, including remote communities, new immigrants, and low-income individuals, as well as First Nations, Inuit, and Métis peoples. Recent data from the Saskatchewan Cancer Screening Program estimated screening rates in northern Saskatchewan communities to be only 18% [3]. Reasons for poor adherence include barriers such as fear or discomfort, embarrassment, and anxiety, as well as limitations of access to care [5].

In Canada, over 40% of all cervical cancers are found in women who have never been screened [6], emphasizing the criticality of increasing the effectiveness and breadth of screening. Saskatchewan has the lowest percentage of cervical cancers diagnosed at Stage 1 in the country [4], the earliest and most prognostically favourable stage of the disease. This is likely due to the higher proportion of unscreened or under-screened individuals in the province, as these early stages are less likely to present with symptoms and are instead identified upon screening. 

HPV, a sexually transmitted infection, has been identified as the cause of over 90% of all cervical cancers [7]. HPV testing is more sensitive in detecting high risk for cervical cancer than PAP tests [8] and is a cost-effective alternative to traditional PAP tests [9,10]. Many Canadian provinces have begun the transition to primary HPV screening for cervical cancer in order to meet the goal of eliminating cervical cancer by 2040 as set out by the World Health Organization (WHO) and per the Canadian Partnership Against Cancer (CPAC) Action Plan. This plan includes Canada’s transition from traditional PAP testing to HPV primary screening, as well as incorporating self-sampling as part of screening [11]. Saskatchewan updated its cervical cancer screening guidelines in 2023 with the plan to move towards primary HPV screening in the next 5 years. Unlike PAP tests, HPV testing can be done as a self-swab, providing individuals with an alternative screening method that does not require visiting a clinician. Evidence from initiatives in provinces such as Manitoba, where less than 3% of a comparable under-screened population is typically screened, indicates that the use of self-administered HPV testing has the potential to significantly increase HPV screening rates to 9.6% [12]. Similarly, in a comparable study conducted in Ontario, HPV screening response rates rose to 32% from 9% by distributing self-administered HPV testing kits in a rural setting [13]. Furthermore, in a separate Ontario study, under- or never-screened racialized women in an urban area were receptive to HPV self-swabs, with 61% of those in the study completing a swab [14]. In Quebec, there was a 77.5% return rate of mailed HPV self-sampling kits after women opted into testing, with 95.8% of participants indicating they would choose this method for further primary screening [15].

In this pilot study, we aimed to directly target Saskatchewan’s under-screened in both urban and rural populations to assess cervical cancer screening participation rates with HPV self-sampling to determine if this provides an effective alternative to traditional cervical cancer screening in this unique population. 

## 2. Materials and Methods

### 2.1. Setting

The Screening Program for Cervical Cancer (SPCC) at the Saskatchewan Cancer Agency manages the cervical cancer screening registry for the province of Saskatchewan. At the time of this study, SPCC guidelines recommended that all women between the ages of 21 and 69 who have ever been sexually active have a PAP test every 2–3 years. SPCC sends reminder letters to women province-wide who are due for their screening every 2 years, unless they have opted out of screening by notifying the SPCC. 

### 2.2. Study Design and Population

This study used a prospective cohort design with *n* = 500 participants who were poorly screened or unscreened. This sample size was informed by the response rate of comparable studies. Specifically, in a very similar population, investigators observed a response rate of 9.6% in a sample size of 500 [12]. Participants were between the ages of 30 and 69 years of age, held valid Saskatchewan health coverage for at least 5 years, had a cervix, and dwelled in northern rural communities or Saskatoon’s inner city and urban geographic areas, as indicated by postal code. Subjects were excluded if they had a history of gynaecologic malignancy, hysterectomy, a record of having received a PAP test in the last 4 years, or lived outside the target geographic areas. Participants were mailed or handed a package containing an informational letter, consent form, instruction guide, swab, biohazard bag, response form and return envelope, and an HPV swab and were instructed on how to complete the swab. The HPV self-sampling swab used in the study was the FlOQSwab^®^ (Copan Diagnostics, Murrieta, CA, USA), a Health Canada-approved medical device. 

The participants were divided into two cohorts, namely, *n* = 250 participants to whom self-swabbing kits were mailed with instructions (Group 1) and *n* = 250 participants to whom the kits were handed out in person through community clinics (Group 2) as an alternative to a PAP test.

Group 1 participants were selected from the cervical cancer screening registry in Saskatchewan. The registry automatically generated a list of the next 250 individuals to whom reminder letters would have been sent. These individuals had no record of PAP screening in the last 4 years and met the geographic restriction. These participants were mailed the standard reminder letter, as well as an option to participate in the study with the self-sampling kit (included in the mailing) through Canada Post. Group 2 consisted of 250 subjects who met the same criteria for being under-screened (no PAP in 4 years or more) but were otherwise visiting a primary care clinic in person for any reason and were offered the self-sample as an option for screening. A total of five primary care clinics for Group 2 were selected: Westside Clinic, Île-à-la-Crosse, La Ronge, Meadow Lake, and Westwinds. Two clinics are in Saskatoon (Westside Clinic and Westwinds) where a higher proportion of under-screened individuals were expected (inner city, new immigrants, trans-gender, sex trade workers), and three medical clinics are located in northern rural communities (Île-à-la-Crosse, La Ronge, Meadow Lake). 

Completed self-sampling kits from both groups were sent for analysis to the Department of Pathology and Laboratory Medicine at the Royal University Hospital in Saskatoon. Swab specimens were eluted into PreservCyt^®^ (Hologic Inc., Marlborough, MA, USA) media. The specimens were analyzed with the Roche cobas^®^ 4800 System (Roche Molecular Systems, Branchburg, NJ, USA) using the cobas^®^ 4800 HPV Test.

Follow-ups and the communication of test results were coordinated by the study team with both a phone call as well as a letter to the participant and their family physician if they consented to having their results forwarded to their care provider. Participants were sent a letter communicating their results, regardless of a positive or negative HPV test. Referrals for colposcopy for those with positive HPV results were then arranged by the study team per standard of care.

### 2.3. Outcomes

This study was designed to examine the potential of using self-administered swabs to increase cervical cancer screening rates in Saskatchewan. The primary outcome was screening participation in under-screened women. The secondary outcome was HPV positivity. We also examined whether the method of the distribution of the self-swab kit, as well as location of the clinic (urban or rural), had an effect on screening participation. 

### 2.4. Statistical Analyses

Descriptive statistics were used to summarize patient characteristics (Table 1) and HPV results. Fisher’s exact tests were used to evaluate whether the proportion of participants with specific outcomes of interest was different between subject groups and stratified sub-groups, including gender, race, and location in an exploratory analysis. Participants with no HPV result (Table 2 “no result”) were combined with negative HPV results for the purposes of statistical testing. A second confirmatory analysis was also conducted that excluded samples with no result. This pilot study was approved by the University of Saskatchewan Health Research Ethics Board (No.: Bio3299), Saskatchewan Cancer Agency data acquisition committee, and Roche Diagnostics).

## 3. Results

From October 2022–March 2023, 250 self-sample kits were mailed out and 250 kits were handed out at various clinics. Of these, 28 individuals responded to the mailed-out kits (screening rate of 11.2%) as compared to the 52 individuals who received a handout kit (20.8%) (Table 1). This difference was statistically significant (*p*-value = 0.005). Mailout responders reported being predominantly Caucasian in ethnicity, while handout responders reported being more evenly distributed among ethnic groups. Among the clinics handing out self-sample kits, Westside Clinic had the highest screening response rate (19/50 = 38%), followed by La Ronge (12/50 = 24%), Île-à-la-Crosse (10/50 = 20%), Westwinds/Saskatoon (8/50 = 16%), and Meadow Lake (3/50 = 6%) (Table 1).

Differences in positivity rates for specific HPV strains or groups of strains measured in each distribution group did not achieve statistical significance (Table 2). There was a numerically higher rate of positivity for participants in the handout population to have at least one strain of HPV (31% vs. 14%). Stratifying further by the location of the clinic, we found that participants seen at Westside Clinic, an inner-city clinic, had the highest rate of respondents, 19 out of 50 (38%). In addition, these participants presented the highest rate of HPV positivity, 9 out of 19 (47.4%) (Table 3). This being said, these differences could not be assigned to any specific strain or group of strains. Indeed, no single strain of high-risk HPV was disproportionally represented in the participants seen at Westside Clinic: HPV 16 (*p* = 0.084), HPV 18 (*p* = 0.054), and other HR HPV (*p* = 0.19).

The results were stratified once again according to race and presented in Table 4. The latter further confirms that while other ethnic groups responded similarly regardless of the delivery method, the self-reported Indigenous and Métis participants were overrepresented in the in-person handout group. The largest proportion of these respondents were from a rural community. Differences in HPV positivity rates between Indigenous and Métis participants, as compared to participants from all other ethnic groups, did not reach statistical significance.

## 4. Discussion

The objective of this pilot study was to assess how HPV self-sampling might improve cervical cancer screening participation rates in Saskatchewan’s under-screened population in both urban and rural settings. With Saskatchewan being one of most sparsely populated provinces, a key motivator was assessing how population density and demographics might lead to adoption rates that differ significantly from those obtained in more densely populated provinces such as British Columbia (BC) or Ontario. Encouraged by the results obtained from using mailout delivery of self-swab kits in Manitoba, a neighboring province with a comparable demographic, this project uniquely explored additional dimensions of HPV self-sampling by also assessing the effectiveness of alternative delivery of these kits by physician hand-out as well as the impact of race. Interestingly, the current study’s overall response rate (~16%) was substantially better than that obtained in the comparably sparse Manitoban population. We attribute this difference mainly to the higher effectiveness of the provider handout arm (~21% of responders) in contrast to the mailout where the response rate (~11% of responders) was comparable to that of the Manitoba study (~10%) despite the latter having a larger sample size. Indeed, the response rate to the care provider handouts was double that of the mailout response, aligning with the similar results reported in a review and meta-analysis of 33 comparable trials [16]. The underlying reasons voiced by the authors mirrored our own interpretations of this phenomenon, namely, highly motivated care providers help overcome several barriers to adoption and increase a subject’s confidence in their ability to self-administer the test by giving verbal instruction on its proper use and interactively answering questions and addressing concerns. In addition, participants presenting to clinics may be inherently more motivated to screen than the mailout participants. While a cost–benefit analysis is beyond the scope of this study, one might appreciate that there are fewer kits lost in the in-person handout method, translating to lower cost. Perhaps an alternative strategy to proactive mailout for reaching remote rural residents would be to mail out upon online request. This has very recently been successfully implemented as an option for cervical cancer screening in British Columbia. 

With regard to screening positivity, responders to the direct handout of self-swab kits presented with numerically higher HPV positivity overall (*p*~0.17), though this did not reach statistical significance. The Westside clinic, an inner-city location, reported the highest number of self-administered swab kits handed out directly by care providers, and these same participants presented a significantly higher positivity rate to at least one HPV strain as compared to all other clinics and mailouts combined. Though the self-reported Indigenous and Métis participants were overrepresented in the handout population, they were not overrepresented at the Westside site as compared to other ethnic groups. These preliminary results suggest that participants living in the inner city may represent a higher risk group, regardless of ethnicity, and more importantly, that the in-person handout of self-sampling kits by a care provider may be an effective way of improving the screening rates in this population. While HPV positivity rates in urban and rural populations are typically comparable [17], the demographic presenting to this particular inner-city clinic (Westside) consisted disproportionately of individuals with no fixed address, suspected substance abuse issues, and several other risk factors, making this group a specific sub-population of urban dwellers. Admittedly, given that this is a pilot study, statistical significance should be interpreted with caution as these analyses are exploratory. 

While the results of this pilot study are admittedly based on a limited sample size, we argue that they nonetheless provide a convincing justification for further study. Specifically, we may want to target inner city populations with a handout method, as this appears to be more effective in these individuals. Likewise, a greater emphasis is still required to reach those rural and northern participants who do not typically respond to mailouts. As much of the population are non-responders, further exploration into the gaps in education and outreach is required, as knowledge, translation, and communication methods can differ for different communities and cultures. Logistical challenges also exist, as mailouts rely on the accuracy of addresses held by the screening program, which are sourced from Saskatchewan healthcare, which relies on individuals maintaining the correct address. Some of these addresses may have been out of date, with letters not reaching the intended participants, increasing the non-responder rate. Moreover, in this first analysis, rural vs. urban location was limited to the location of the clinic in the handout group, not to the address of the participant. In addition to including a larger sample size supporting more robust statistics, a future study of this population would be further informed by added geographical resolution in the mailout group and more granular analysis of non-responder behavior. For example, protocols in place for this study did not enable the investigators to compare mailout response rates in the immediate vicinity of the handout locations. Similarly, it was not possible to track the number of non-responders that subsequently sought out a standard-of-care PAP test, though this is typically low (~3%). Ongoing efforts by our group hope to address these issues in a subsequent phase of study. These measures include the use of a secure web-based system for the self-directed request of sample kits and added molecular resolution in differentiating specific HPV strains. Regardless of the delivery method, one of the main challenges of implementing mail-in or handout strategies will require that it be integrated centrally into the screening program. This constitutes an organizational challenge, including additional outreach, educational resources, and infrastructure. Collaboration and partnership between Saskatchewan Cancer Agency, researchers, behavioral and implementation scientists, policy makers and government officials is key to this process. Ultimately, the results of this pilot study arrive at the same conclusions reported in most, if not all, prior studies, namely, that self-administered HPV sample kits, both provided as in person and as mailouts, are of significant value in improving screening rates and that they should be implemented as a critical part of the standard of care in cancer prevention. 

## 5. Conclusions

These preliminary results of this pilot study suggest that women living in the inner-city locations of Saskatchewan may represent a higher risk group and that the in-person handout of self-sampling may be an effective way of improving screening rates in this population presenting with significantly higher HPV positivity rates. However, mailout kits may also provide some benefit for reaching participants who are not seen in clinics.

## Figures and Tables

**Table 1:** Characteristics of subjects and response rates.

		*n*	All Pts	Handout Responders	Mailout Responders	*p*-Value
*n* (%)			80	52 (20.8%)	28 (11.2%)	0.0005
Gender	*n* (%) Female No Response	80	67 (83.8) 13 (16.3)	43 (82.7) 9 (17.3)	24 (85.7) 4 (14.3)	
Race	*n* (%) Caucasian Indigenous ** Métis Chinese No Response	80	30 (37.5) 18 (22.5) 16 (20.0) 1 (1.3) 15 (18.8)	13 (25.0) 14 (26.9) 14 (26.9) 1 (1.9) 10 (19.2)	17 (60.7) 4 (14.3) 2 (7.1) 0 (0.0) 5 (17.9)	0.003 *
Location *n* (%)	Rural Urban Unknown ^¥^	80	25 (31.3) 27 (33.8) 28 (35.0)	25 (48.1) 27 (51.9) 0 (0.0)	NA NA 28 (100.0)	
Specific Location *n* (%)	Mailout Westside Clinic Île-à-la-Crosse La Ronge Meadow Lake Westwinds	80		19 (23.8) 10 (12.5) 12 (15.0) 3 (3.8) 8 (10.0)	28 (35.0) NA	

* Test is Caucasian versus others; ** Ethnicity was self-reported. However, the term Indigenous may have been interpreted by the respondent as including First Nation, Métis, and Inuit peoples. ^¥^ Rural vs. urban location was limited to the location of the clinic in the handout group, not the address of the participant (all mailouts (28) were assigned as unknown).

**Table 2:** Characteristics of Participants including cobas^®^ 4800 HPV Test positivity.

		*n*	All Pts	Handout Responders	Mailout Responders	*p*-Value
Other HR HPV	*n* (%) Negative Positive ^†^ No Results *	80	59 (73.8) 16 (20.0) 5 (6.3)	37 (71.2) 12 (23.1) 3 (5.8)	22 (78.6) 4 (14.3) 2 (7.1)	0.40 ^#^
HPV 16	*n* (%) Negative Positive No Results	80	68 (85.0) 5 (6.3) 7 (8.8)	44 (84.6) 4 (7.7) 4 (7.7)	24 (85.7) 1 (3.6) 3 (10.7)	0.65 ^#^
HPV 18	*n* (%) Negative Positive No Results	80	72 (90.0) 2 (2.5) 6 (7.5)	47 (90.4) 2 (3.9) 3 (5.8)	25 (89.3) 0 (0.0) 3 (10.7)	0.54 ^#^
Any HPV	*n* (%) Negative Positive No Results	80	55 (68.8) 20 (25.0) 5 (6.3)	33 (63.5) 16 (30.8) 3 (5.8)	22 (78.6) 4 (14.3) 2 (7.1)	0.17 ^#^

^†^ Positive if any one of cobas^®^ 4800 HPV test results was positive. Negative if no result was positive and at least one result was negative. No results only if all three cobas^®^ HPV test results were listed as no results. * Results were similar if we excluded the “no result” subjects (*p*-values of 0.35, 0.24, 0.71, and 0.17 for HPV–Other, −16, −18 and Any HPV, respectively); ^#^ test is positive vs. negative/no result.

**Table 3:** Cobas^®^ HPV status of handout participants according to location.

		Handout			
		Westside Clinc	Île-à-la-Crosse	La Ronge	Other
Responses		19	10	12	11
*n* Forms		50	50	50	100
Screened (%)		38%	20%	24%	11%
Other HR HPV *n* (%)	Negative Positive No Results	12 (63.2) 6 (31.6) 1 (5.3)	7 (70.0) 2 (20.0) 1 (10.0)	9 (75.0) 2 (16.7) 1 (8.3)	9 (81.8) 2 (18.2) 0
HPV 16 *n* (%)	Negative Positive No Results	14 (73.7) 3 (15.8) 2 (10.5)	9 (90.0) 0 1 (10.0)	10 (83.3) 1 (8.3) 1 (8.3)	11 (100) 0 0
HPV 18 *n* (%)	Negative Positive No Results	16 (84.2) 2 (10.5) 1 (5.3)	9 (90.0) 0 1 (10.0)	11 (91.7) 0 1 (8.3)	11 (100) 0 0
Any HPV *n* (%)	Negative Positive No Results ^†^	9 (47.4) 9 (47.4) 1 (5.3)	7 (70.0) 2 (20.0) 1 (10.0)	8 (66.7) 3 (25.0) 1 (8.3)	9 (81.8) 2 (18.2) 0

^†^ No results only if all three cobas^®^ HPV test results were listed as no results.

**Table 4:** Characteristics of subjects and responses by race.

		*n*	Indigenous/MÉTIS Participants	Other Participants	*p*-Value
*n*			34 (42%)	46 (58%)	
Gender	*n* (%) Female No Response	80	29 (85.3) 5 (14.7)	38 (82.6) 8 (17.4)	1.00
Race	*n* (%) Caucasian Indigenous Métis Chinese No Response	80	0 18 (52.9) 16 (47.1) 0 0	30 (65.2) 0 0 1 (2.2) 15 (32.6)	Not calculated
Other HR HPV	*n* (%) Negative Positive No Results	80	26 (76.5) 5 (14.7) 3 (8.8)	33 (71.7) 11 (23.9) 2 (4.4)	0.40 ^#^
HPV 16	*n* (%) Negative Positive No Results	80	28 (82.4) 3 (8.8) 3 (8.8)	40 (87.0) 2 (4.4) 4 (8.7)	0.65 ^#^
HPV 18	*n* (%) Negative Positive No Results	80	30 (88.2) 1 (2.9) 3 (8.8)	42 (91.3) 1 (2.2) 3 (6.5)	1.00 ^#^
Any HPV Positive	*n* (%) Negative Positive No Results ^†^	80	23 (67.7) 8 (23.5) 3 (8.8)	32 (69.6) 12 (26.1) 2 (4.4)	1.00 ^#^
Mailout Type	*n* (%) Handout Mailout	80	28 (82.4) 6 (17.7)	24 (52.2) 22 (47.8)	0.009
Location	*n* (%) Rural Urban Unknown ^¥^	80	18 (52.9) 10 (29.4) 6 (17.7)	7 (15.2) 17 (37.0) 22 (47.8)	<0.001 Rural vs. other
Specific Location	*n* (%) Mailout Westside Île-à-la-Crosse La Ronge Meadow Lake Westwinds	80	6 (17.7) 9 (26.4) 14 (29.4) 7 (20.6) 1 (2.9) 1 (2.9)	22 (47.7) 10 (21.7) 0 5 (10.9) 2 (4.4) 6 (13.0)	

^†^ Positive if any one of cobas^®^ 4800 HPV test results was positive. Negative if no result was positive and at least one result was negative. No results only if all three cobas^®^ 4800 HPV test results were listed as no results. ^#^ test is positive vs. negative/no result. ^¥^ Rural vs. urban location was limited to the location of the clinic in the handout group, not the address of the participant (all mailouts (28) were assigned as unknown).

## Data Availability

The data presented in this study are available on request from the corresponding author due to privacy and ethical reasons.
